# Ionic mechanisms of ST segment elevation in electrocardiogram during acute myocardial infarction

**DOI:** 10.1186/s12576-020-00760-3

**Published:** 2020-07-13

**Authors:** Jun-ichi Okada, Katsuhiko Fujiu, Kazunori Yoneda, Takashi Iwamura, Takumi Washio, Issei Komuro, Toshiaki Hisada, Seiryo Sugiura

**Affiliations:** 1 UT-Heart Inc., 3-25-8 Nozawa, Setagaya, Tokyo, 154-0003 Japan; 2 Future Center Initiative, The University of Tokyo, 178-4-4 Wakashiba, Kashiwa, Chiba 277-0871 Japan; 3 Department of Cardiovascular Medicine, Graduate School of Medicine, The University of Tokyo, Bunkyo, Tokyo 113-8655 Japan; 4 Department of Advanced Cardiology, Graduate School of Medicine, The University of Tokyo, Bunkyo, Tokyo 113-8655 Japan; 5 Healthcare Solutions Unit, Fujitsu Limited, Minato, Tokyo 108-0075 Japan

**Keywords:** Myocardial infarction, ST elevation, Simulation, Solid angle

## Abstract

ST elevation on an electrocardiogram is a hallmark of acute transmural ischemia. However, the underlying mechanism remains unclear. We hypothesized that high ischemic sensitivities of epicardial adenosine triphosphate-sensitive potassium (IK_ATP_) and sodium (INa) currents play key roles in the genesis of ST elevation. Using a multi-scale heart simulation under moderately ischemic conditions, transmural heterogeneities of IK_ATP_ and INa created a transmural gradient, opposite to that observed in subendocardial injury, leading to ST elevation. These heterogeneities also contributed to the genesis of hyper-acute T waves under mildly ischemic conditions. By contrast, under severely ischemic conditions, although action potentials were suppressed transmurally, the potential gradient at the boundary between the ischemic and normal regions caused ST elevation without a contribution from transmural heterogeneity. Thus, transmural heterogeneities of ion channel properties may contribute to the genesis of ST–T changes during mild or moderate transmural ischemia, while ST elevation may be induced without the contribution of heterogeneity under severe ischemic conditions.

## Background

Despite its long history, the electrocardiogram (ECG) remains an indispensable tool for diagnosis of heart diseases such as arrhythmias and myocardial ischemia. In particular, elevation of the ST segment is a hallmark of acute myocardial infarction, and helps to identify patients in a critical condition. Acidosis, anoxia, and elevated extracellular potassium concentration (hyperkalemia) induced by ischemia can cause an elevation in the resting potential, with shortening of the action potential duration and decreased amplitude.

In cases of angina pectoris, in which ischemia usually occurs in the subendocardial region of the ventricular wall, the developed potential gradient generates the current of injury flowing to the normal epicardial area during diastole, resulting in a positive potential of the TQ-segment of the ECG. During systole, depolarization of both the ischemic and normal myocardium cancels the potential gradient between these areas, causing the potential of the ST segment to become zero. However, because of conventions in ECG recording, which sets the TQ-segment at zero, the ST segment is regarded as negative [12, 24]. Accordingly, ST segment elevation during acute myocardial infarction requires the injury current to flow in the opposite direction [12, 13, 24], which can be caused by greater depression of the epicardial action potential. However, despite their favorable anatomical location for coronary perfusion, the severe effects on epicardial cells suggest that they should exhibit properties sensitive to ischemia. Indeed, distinct properties of sodium and adenosine triphosphate (ATP)-sensitive potassium channels have been reported in epicardial cells [4, 8], although the contributions of these channels to the genesis of ST elevation are difficult to evaluate in vivo.

Computer simulation is a useful tool for basic and clinical studies of cardiac electrophysiology [16, 33]. In particular, multi-scale simulation models provide a unique opportunity to examine the relationship between microscopic findings and clinical observations. We previously developed a multi-scale model of the human heart and torso, which can reproduce the surface ECG based on the ionic currents of cell models of electrophysiology [19–23, 32]. In the present study, we applied this heart model to examine the ionic mechanism of ST elevation during acute myocardial infarction. In particular, we examined the nature of the injury current, including the possible involvement of transmural heterogeneity of sodium and ATP-sensitive potassium channels. We tested multiple scenarios to find that although heterogeneity in the properties of these channels plays a role during mild ischemia, ST elevation during severe ischemia can occur without its involvement.

## Methods

### Simulation model of human ventricles and torso

**Fig. 1:**
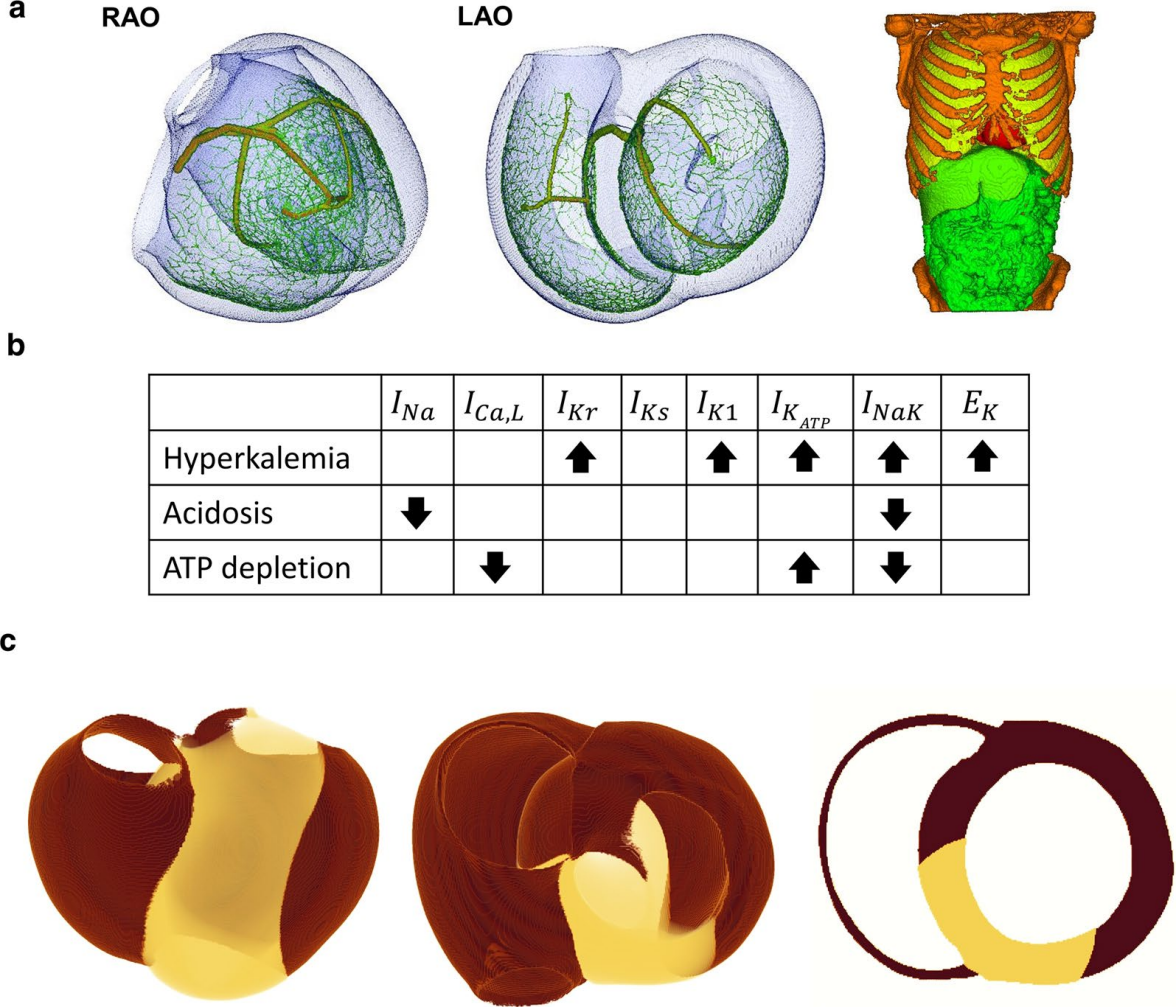
Heart and torso meshes. **a** Left: heart meshes with the conduction system in the right anterior oblique (RAO) and left anterior oblique (LAO) views. Right: torso mesh. **b** Effects of hyperkalemia, acidosis, and adenosine triphosphate (ATP) depletion on sodium ($I_{\text{Na}}$), L-type calcium ($I_{{{\text{Ca}},{\text{L}}}}$), rapid delayed rectifier potassium ($I_{\text{Kr}}$), slow delayed rectifier potassium ($I_{\text{Ks}}$), inward rectifier potassium ($I_{{{\text{K}}1}}$), ATP-sensitive potassium ($I_{{{\text{K}}_{\text{ATP}} }}$), and sodium/potassium ATPase (I_NaK_) currents, and the reversal potential for potassium currents ($E_{K}$). Upward arrow: facilitatory effect. Downward arrow: Inhibitory effect. (**c**) Front (left), top (middle), and short axis (right) views of the heart model. The ischemic condition was applied to the pale-colored region perfused by the anterior descending artery

We used the finite element method models of healthy adult human ventricles and torso that we previously reported and validated [20, 22, 23]. Briefly, the geometries of these models were based on the multidetector computerized tomography data of a healthy volunteer, and were subdivided into 244,187,136 and 40,038,400 voxels for the heart and torso, respectively (Fig. 1a). We applied the cell model of cardiomyocytes with different electrophysiological properties (endocardial, mid-myocardial [M], and epicardial) [17] to the appropriate finite element method nodes of the ventricular wall, with some modifications. As previously reported [23, 26], the equations for the activation gate (m) of the sodium channel were replaced by those of ten Tusscher’s model [29] to reproduce the physiological conduction velocity. Endocardial, M, and epicardial cells were distributed at 0–25%, 25–70%, and 70–100% of the wall thickness from the endocardial side [22, 23].

We mapped previously reported human data on the spatial orientation of the myocyte (fiber orientation) to our model using the rule-based method [9]. We also modeled the conduction system using a one-dimensional network consisting of the free-running (insulated) part connecting the atrioventricular node to the sites of earliest activation, and the network spreading from these sites along the endocardial surface. The electrophysiological properties were reproduced using the cell model proposed by Stewart et al. [28]. The propagation of excitation was formulated with the bidomain model, and was solved using the parallel multilevel technique, which we previously developed and validated [31]. Briefly, in the heart domain

$$\beta \left( {C_{m} \frac{{\partial V_{m} }}{{\partial {\text{t}}}} + I_{{{\text{ion}}}} } \right) = I_{{{\text{stim}}}} + \frac{\partial }{{\partial x_{i} }}\left( { G_{{ij}}^{I} \frac{{\partial \theta{ ^{{\text{I}}}} }}{{\partial x_{j} }}} \right),$$

$${{\beta }}\left( {C_{m} \frac{{\partial V_{m} }}{\partial t} + I_{\text{ion}} } \right) = I_{\text{stim}} - \frac{\partial }{{\partial x_{i} }}\left( {G_{ij}^{\text{E}} \frac{{\partial \emptyset^{\text{E}} }}{{\partial x_{j} }}} \right),$$

where $\emptyset^{\text{I}}$ and $\emptyset^{\text{E}}$ are the intracellular and extracellular potentials, respectively, $V_{m}$ is the membrane potential defined as $V_{m} = \emptyset^{\text{I}} - \emptyset^{\text{E}}$, $\beta$ is the surface-to-volume ratio of the tissue (2000 cm^−1^), $C_{m}$ is the membrane capacitance (= 1 ${{\mu} \text{F}}/{\text{cm}}^{2}$), t is time, $G_{ij}^{E}$ and $G_{ij}^{I}$ are the extracellular and intracellular conductivity tensors, respectively, accounting for the anisotropy of the cardiac tissue, $I_{\text{stim}}$ is the stimulation current, $I_{\text{ion}}$ is the sum of the ionic transmembrane currents calculated using the cell model, and the indices *i* and *j* vary from 1 to 3. In the torso domain, we solved the following Laplace’s equation:

$$\frac{\partial }{{\partial x_{i} }}\left( {G_{ij}^{T} \frac{{\partial \emptyset^{\text{T}} }}{{\partial {\text{x}}_{\text{j}} }}} \right) = 0,$$

where $\emptyset^{\text{T}}$ is the potential, while $G_{ij}^{\text{T}}$ is the isotropic conductivity at each point and differs in each organ. The following conditions were imposed on the boundaries between the domains:

$$n_{i} \left( {G_{ij}^{\text{T}} \frac{{\partial \emptyset^{\text{T}} }}{{\partial x_{j} }}} \right) = 0$$

on the boundary of the torso domain, and

$$n_{i} \left( {G_{ij}^{\text{E}} \frac{{\partial \emptyset^{\text{E}} }}{{\partial x_{j} }}} \right) = n_{i} \left( {G_{ij}^{\text{T}} \frac{{\partial \emptyset^{\text{T}} }}{{\partial x_{j} }}} \right)\,\, {\text{and }}\,\, \emptyset^{\text{E}} = \emptyset^{\text{T}}$$

on the boundary of the heart domain. The reference point for the extracellular potential was placed at the bottom right of the torso model. The parameter values used are listed in Table 1.

**Table 1:** Conductivity of organs for simulation

Organ	Conductivity (mS/cm)
Heart (intracellular)	
Fiber direction	6.2
Fiber normal direction	2
Heart (extracellular)	
Fiber direction	4
Fiber normal direction	2
Blood	7.8
Atrium, artery, vein	3
Muscle	2.56
Lung	0.83
Esophagus, stomach, bowel	2
Spleen, liver, kidney	1.67
Bone	0.1
Fat	0.5
Skin, body surface	40

### Channel models sensitive to ischemic conditions

Anoxia caused by ischemia induces a variety of metabolic changes including acidosis, ATP depletion, and hyperkalemia. The influences of these changes on ionic currents were modeled as follows (Fig. 1b).

#### Hyperkalemia

The following formulations on the effects of hyperkalemia in the O’Hara Rudy (ORd) model [17] were adopted.

##### Reversal potentials

Reversal potentials for the rapid delayed rectifier potassium ($I_{\text{Kr}}$) and inward rectifier potassium ($I_{{{\text{K}}1}}$) currents:

$${E_{K}} = \frac{\text {RT}}{{\text{F}}}{\text {ln}}\left( {\frac{\left[ {\text{K}}{^+} \right]_{\text{o} }}{\left[ {\text{K}}{^+}\right]_{i} }} \right)$$

Reversal potential for the slow delayed rectifier potassium ($I_{\text{Ks}}$):

$$E_{{{\text{Ks}}}} = \frac{{{\text{RT}}}}{{\text{F}}}\ln \left( {\frac{{\left[ {{\text{K}}^{ + } } \right]_{o} + {\text{PR}}_{{{\text{Na,K}}}} \cdot \left[ {{\text{Na}}^{ + } } \right]_{{\text{o}}} }}{{\left[ {{\text{K}}^{ + } } \right]_{i} + {\text{PR}}_{{{\text{Na,K}}}} \cdot \left[ {{\text{Na}}^{ + } } \right]_{{\text{i}}} }}} \right),$$

$${\text{PR}}_{{{\text{Na}},{\text{K}}}} = 0.01833.$$

Body temperature (T) was assumed to be 310 K.

Conductance of the rapid delayed rectifier potassium current (*I*_Kr_):

$$G_{\text{Kr}} = 0.046 \cdot \sqrt {\frac{{\left[ {{\text{K}}^{ + } } \right]_{\text{o}} }}{5.4}} \left[ {{\text{mS}}/{{\upmu F}}} \right].$$

Conductance of the inward rectifier potassium current (I_K1_):

$$G_{{{\text{K}}1}} = 0.1908 \cdot \sqrt {\left[ {{\text{K}}^{ + } } \right]_{\text{o}} } \left[ {{\text{mS}}/{{\upmu F}}} \right].$$

#### ATP depletion

##### Sodium/potassium ATPase current (I_NaK_)

The I_NaK_ current in the ORd model is dependent on [MgATP], $\left[ {{\text{H}}^{ + } } \right]$, $\left[ {{\text{Na}}^{ + } } \right]$, and $\left[ {{\text{K}}^{ + } } \right]$. Although it was previously reported that intracellular acidosis inhibits I_NaK_ [30], [H^+^] is not explicitly defined as intracellular in the ORd model. In the present study, we assumed that $\left[ {{\text{H}}^{ + } } \right]$ was in a quasi-equilibrium between the intracellular and extracellular spaces. Thus, we used the same values for the intracellular and extracellular spaces.

$$\begin{gathered} K_{{{\text{Nai}}}} = 9.073 \cdot \exp \left( {\frac{{ - 0.1550 \cdot {\text{V}} \cdot {\text{F}}}}{{3 \cdot {\text{R}} \cdot {\text{T}}}}} \right), \hfill \\ {\text{K}}_{{{\text{Nao}}}} = 22.78 \cdot {\text{exp}}\left( {\frac{{1.1550 \cdot {\text{V}} \cdot {\text{F}}}}{{3 \cdot {\text{R}} \cdot {\text{T}}}}} \right) \hfill \\ \end{gathered} ,$$

$$\left[ {\text{P}} \right] = {{\left[ {{{\varSigma P}}} \right]} \mathord{\left/ {\vphantom {{\left[ {{{\varSigma P}}} \right]} {\left( {1 + \frac{{\left[ {{\text{H}}^{ + } } \right]}}{{1.687 \cdot 10^{ - 7} }} + \frac{{\left[ {{\text{Na}}^{ + } } \right]_{\text{i}} }}{224} + \frac{{\left[ {{\text{K}}^{ + } } \right]_{\text{i}} }}{292}} \right)}}} \right. \kern-0pt} {\left( {1 + \frac{{\left[ {{\text{H}}^{ + } } \right]}}{{1.687 \cdot 10^{ - 7} }} + \frac{{\left[ {{\text{Na}}^{ + } } \right]_{\text{i}} }}{224} + \frac{{\left[ {{\text{K}}^{ + } } \right]_{\text{i}} }}{292}} \right)}},$$

$$\alpha_{1} = \frac{{949.5\left( {\frac{{\left[ {{\text{Na}}^{ + } } \right]_{\text{i}} }}{{{\text{K}}_{\text{Nai}} }}} \right)^{3} }}{{\left( {1 + \frac{{\left[ {{\text{Na}}^{ + } } \right]_{\text{i}} }}{{{\text{K}}_{\text{Nai}} }}} \right)^{3} + \left( {1 + \frac{{\left[ {{\text{K}}^{ + } } \right]_{\text{i}} }}{0.5}} \right)^{2} - 1}},$$

$$\beta_{1} = 182.4 \cdot \left[ {\text{MgADP}} \right],$$

$$\alpha_{2} = 687.2,$$

$$\beta_{2} = \frac{{39.4\left( {\frac{{\left[ {{\text{Na}}^{ + } } \right]_{\text{o}} }}{{{\text{K}}_{\text{Nao}} }}} \right)^{3} }}{{\left( {1 + \frac{{\left[ {{\text{Na}}^{ + } } \right]_{\text{o}} }}{{{\text{K}}_{\text{Nao}} }}} \right)^{3} + \left( {1 + \frac{{\left[ {{\text{K}}^{ + } } \right]_{\text{o}} }}{0.3582}} \right)^{2} - 1}},$$

$$\alpha_{3} = \frac{{1899\left( {\frac{{\left[ {{\text{K}}^{ + } } \right]_{\text{o}} }}{{{\text{K}}_{\text{Ko}} }}} \right)^{2} }}{{\left( {1 + \frac{{\left[ {{\text{Na}}^{ + } } \right]_{\text{o}} }}{{{\text{K}}_{\text{Nao}} }}} \right)^{3} + \left( {1 + \frac{{\left[ {{\text{K}}^{ + } } \right]_{\text{o}} }}{0.3582}} \right)^{2} - 1}},$$

$$\beta_{3} = \frac{{79300 \cdot \left[ {\text{P}} \right] \cdot \left[ {{\text{H}}^{ + } } \right]}}{{1 + \frac{{\left[ {\text{MgATP}} \right]}}{{1.698 \cdot 10^{ - 7} }}}},$$

$$\alpha_{4} = \frac{{639 \cdot \frac{{\left[ {\text{MgATP}} \right]}}{{1.698 \cdot 10^{ - 7} }}}}{{1 + \frac{{\left[ {\text{MgATP}} \right]}}{{1.698 \cdot 10^{ - 7} }}}},$$

$$\beta_{4} = \frac{{40\left( {\frac{{\left[ {{\text{K}}^{ + } } \right]_{\text{i}} }}{0.5}} \right)^{2} }}{{\left( {1 + \frac{{\left[ {{\text{Na}}^{ + } } \right]_{\text{i}} }}{{{\text{K}}_{\text{Nai}} }}} \right)^{3} + \left( {1 + \frac{{\left[ {{\text{K}}^{ + } } \right]_{\text{i}} }}{0.5}} \right)^{2} - 1}},$$

$$x_{1} = \alpha_{4} \cdot \alpha_{1} \cdot \alpha_{2} + \beta_{2} \cdot \beta_{4} \cdot \beta_{3} + \alpha_{2} \cdot \beta_{4} \cdot \beta_{3} + \beta_{3} \cdot \alpha_{1} \cdot \alpha_{2} ,$$

$$x_{2} = \beta_{2} \cdot \beta_{1} \cdot \beta_{4} + \alpha_{1} \cdot \alpha_{2} \cdot \alpha_{3} + \alpha_{3} \cdot \beta_{1} \cdot \beta_{4} + \alpha_{2} \cdot \alpha_{3} \cdot \beta_{4} ,$$

$$x_{3} = \alpha_{2} \cdot \alpha_{3} \cdot \alpha_{4} + \beta_{3} \cdot \beta_{2} \cdot \beta_{1} + \beta_{2} \cdot \beta_{1} \cdot \alpha_{4} + \alpha_{3} \cdot \alpha_{4} \cdot \beta_{1} ,$$

$$x_{4} = \beta_{4} \cdot \beta_{3} \cdot \beta_{2} + \alpha_{3} \cdot \alpha_{4} \cdot \alpha_{1} + \beta_{2} \cdot \alpha_{4} \cdot \alpha_{1} + \beta_{3} \cdot \beta_{2} \cdot \alpha_{1} ,$$

$$E_{1} = \frac{{x_{1} }}{{x_{1} + x_{2} + x_{3} + x_{4} }},$$

$$E_{2} = \frac{{x_{2} }}{{x_{1} + x_{2} + x_{3} + x_{4} }},$$

$$E_{3} = \frac{{x_{3} }}{{x_{1} + x_{2} + x_{3} + x_{4} }},$$

$$E_{4} = \frac{{x_{4} }}{{x_{1} + x_{2} + x_{3} + x_{4} }},$$

$$I_{\text{NaK}} = 30 \cdot \left( {3 \cdot \left( {E_{1} \cdot \alpha_{3} - E_{2} \cdot \beta_{3} } \right) + 2 \cdot \left( {E_{4} \cdot \beta_{1} - E_{3} \cdot \alpha_{1} } \right)} \right).$$

#### ATP-sensitive potassium current ($I_{{{\text{K}}_{\text{ATP}} }}$)

Because the $I_{{{\text{K}}_{\text{ATP}} }}$ current is not implemented in the ORd model, we adopted the model by Shaw et al. [27], with modifications based on the experimental study on human ventricular cells [1]:

$${\text{IK}}_{\text{ATP}} = G_{{{\text{K}}\left( {\text{ATP}} \right)}} \frac{1}{{1 + \left( {\frac{{\left[ {\text{ATP}} \right]}}{{{\text{Kd}}_{\text{KATP}} }}} \right)^{1} }}\left( {\frac{{\left[ {\text{K}} \right]_{\text{o}} }}{5.4}} \right)^{0.024} \left( {V_{m} - E_{K} } \right),$$

where ${\text{Kd}}_{\text{KATP}}$ = 0.02 mM. For $G_{{{\text{K}}\left( {\text{ATP}} \right)}}$, we adopted the value for the guinea pig ventricular myocyte (0.05 mS/cm^2^) [15], because only the single channel conductance was reported by Babenko et al. [1].

##### L-type calcium current (*I*_Ca,L_)

ATP-dependence of the *I*_Ca,L_ was modeled by Shaw et al. for the guinea pig myocyte [27], as follows:

$$P_{{{\text{Ca}},{\text{L}},{\text{ATP}}}} = \frac{1}{{1 + \left( {\frac{1.4}{{\left[ {\text{ATP}} \right]}}} \right)^{2.6} }}.$$

We multiplied $P_{{{\text{Ca}},{\text{L}},{\text{ATP}}}}$ to ICa,L calculated by the ORd model [17].

#### Acidosis

##### *I*_Na_

Jones et al. [11] examined the pH-dependence of the properties of the human cardiac sodium channel (Na_V_1.5) heterogeneously expressed in Xenopus oocytes, and proposed the following modifications at pH 6.0 to the ventricular cell model by ten Tusscher et al. [29]:

(i) Steady-state activation was scaled by 0.62.
(ii) Steady-state fast inactivation:
  $${\text{h}}_{\infty } = \frac{1}{{\left[ {1 + { \exp }({\text{A}} \cdot \left( {{\text{V}} - {\text{V}}_{1/2} } \right)} \right]}}.$$
  A = 0.178, $${\text{V}}_{1/2}$$ = − 77.16 at pH 7.4.
  A = 0.167, $${\text{V}}_{1/2}$$ = − 73.23 at pH 6.0.
(iii) Steady-state slow inactivation:
  $${\text{h}}_{\infty } = \frac{1}{{\left[ {1 + { \exp }({\text{A}} \cdot \left( {{\text{V}} - {\text{V}}_{1/2} } \right)} \right]}}.$$
  A = 0.136, $${\text{V}}_{1/2}$$ = − 82.66 at pH 7.4.
  A = 0.117, ${\text{V}}_{1/2 }$ = − 83.09 at pH 6.0. ${\text{h}}_{\infty } = 1.02 \cdot {\text{h}}_{\infty }$ at pH 6.0.

##### Maximal conductance of the late sodium current

Maximum conductance of the late sodium current was scaled transmurally by 1.51 at pH 6.0. We made these modifications to ${\text{h}}_{\infty }$ and the maximal conductance of the late sodium current of the ORd model.

##### Transmural heterogeneity in response to ischemia

We found two reports supporting the susceptibility of epicardial cells to ischemia compared with endocardial cells.

(i) ATP-sensitive potassium current (IK_ATP_): Furukawa et al. compared the response of the IK_ATP_ channel to ATP depletion between endocardial and epicardial cells isolated from feline ventricles, and found that the ATP concentration at half-maximal inhibition of the channel was 23.6 ${\mu}{\text{M}}$ for endocardial cells and 97.6 ${\mu}{\text{M}}$ for epicardial cells [8]. To incorporate this transmural heterogeneity, we modified the equation for IK_ATP_ as follows:

$${\text{IK}}_{\text{ATP}} = {\text{G}}_{{{\text{K}}\left( {\text{ATP}} \right)}} \frac{1}{{1 + \left( {\frac{{\left[ {\text{ATP}} \right]}}{{{\text{Kd}}_{\text{KATP}} }}} \right)^{1} }}\left( {\frac{{\left[ {\text{K}} \right]_{\text{o}} }}{5.4}} \right)^{0.024} \left( {{\text{V}}_{\text{m}} - {\text{E}}_{\text{K}} } \right),$$

where ${\text{Kd}}_{\text{KATP}}$ = 0.02 mM for endocardial cells and 0.1 mM for epicardial cells, (ii) Sodium channel current (*I*_Na_): Cordeiro et al. measured the steady-state inactivation of the *I*_Na_ current using the whole cell clamp technique in epicardial and endocardial cells isolated from the canine ventricle [4]. In that study, the 8.1-mV greater negative half-activation voltage of epicardial cells compared with endocardial cells was found to contribute to the higher sensitivity of the epicardial cells to changes in ischemia. We shifted the half-activation voltage of epicardial cells to model this heterogeneity.

### Simulation of ischemia

We modeled a case in which the proximal portion of the left anterior descending artery, just after the bifurcation of the left circumflex artery, was blocked (Fig. 1c). We studied the three types of ischemia in this region:

(i) To examine the mechanisms underlying transmural ischemia, acute subendocardial ischemia was simulated for comparison. In this case, only the endocardial region (0–50% of the wall thickness) was subject to ischemia ([ATP] = 1.0 mM, [*K*]_o_ = 9.0 mM). Considering the ischemia-resistant nature of the Purkinje network [2], the electrophysiological properties of the Purkinje system were not modified.
(ii) For acute transmural ischemia, the ischemic condition was applied transmurally to the anteroseptal region indicated in Fig. 1c. Because the detailed time course of metabolic changes in the clinical settings was unavailable, we repeated the simulations with multiple combinations of ATP concentrations ([ATP]; range, 10–0.1 mM) and extracellular potassium concentrations ([K]_o_; range, 5.4–12 mM). Because of the lack of experimental data, the pH was only set at 6.0. Furthermore, in this case the Purkinje system was made intact.
(iii) For transmural ischemia in the chronic phase, we simulated the old myocardial infarction by eliminating the cellular function in the ischemic region. For each of the normal and ischemic conditions, we repeated 1000 cycles of simulations for the three types of cell models at different [ATP], [K^+^], and pH as the initial condition. The concentrations of the major ions, [ATP], and pH after 1000 cycles of stimulation of epicardial, M, and endocardial cells under normal conditions are summarized in Table 2. Using the final results of the cell simulations as the initial condition, we then performed the heart simulation for five beats. In all simulations, the ECG waveforms from the fourth and fifth beats were superimposable, indicating that the system was in a quasi-steady state. In all simulations, the heart was paced at 1 Hz by applying a stimulus to the root of the His–Purkinje system.

**Table 2:** Cell parameters under normal conditions

Cell type and parameter	Value
Epi cell	
$\left[ {\text{Na}} \right]_{\text{i}}$: intracellular sodium ion concentration [mM]	7.83
$\left[ {\text{Ca}} \right]_{\text{i}}$: intracellular calcium ion concentration [mM]	8.64D-5
$\left[ {\text{K}} \right]_{\text{i}}$: intracellular potassium ion concentration [mM]	140
M cell	
$\left[ {\text{Na}} \right]_{\text{i}}$: intracellular sodium ion concentration [mM]	8.88
$\left[ {\text{Ca}} \right]_{\text{i}}$: intracellular calcium ion concentration [mM]	1.05D-4
$\left[ {\text{K}} \right]_{\text{i}}$: intracellular potassium ion concentration [mM]	139
Endo cell	
$\left[ {\text{Na}} \right]_{\text{i}}$: intracellular sodium ion concentration [mM]	7.06
$\left[ {\text{Ca}} \right]_{\text{i}}$: intracellular calcium ion concentration [mM]	8.58D-5
$\left[ {\text{K}} \right]_{\text{i}}$: intracellular potassium ion concentration [mM]	141
$\left[ {\text{Na}} \right]_{\text{o}}$: extracellular sodium ion concentration [mM]	140
$\left[ {\text{Ca}} \right]_{\text{o}}$: extracellular calcium ion concentration [mM]	2
$\left[ {\text{K}} \right]_{\text{o}}$: extracellular potassium ion concentration [mM]	5.4
[ATP]: ATP concentration [mM]	9.8
pH	7.4

ATP, adenosine triphosphate

### Computation

Computation was performed using a HP Blade System c7000 (Intel Xeon E5-2670 [2.6 GHz]), and the computational time was 3 h for a single beat with 254 cores. All program codes were written in-house and validated in our previous studies [19, 20, 22, 23].

## Results

### ECG under normal conditions and subendocardial ischemia

**Fig. 2:**
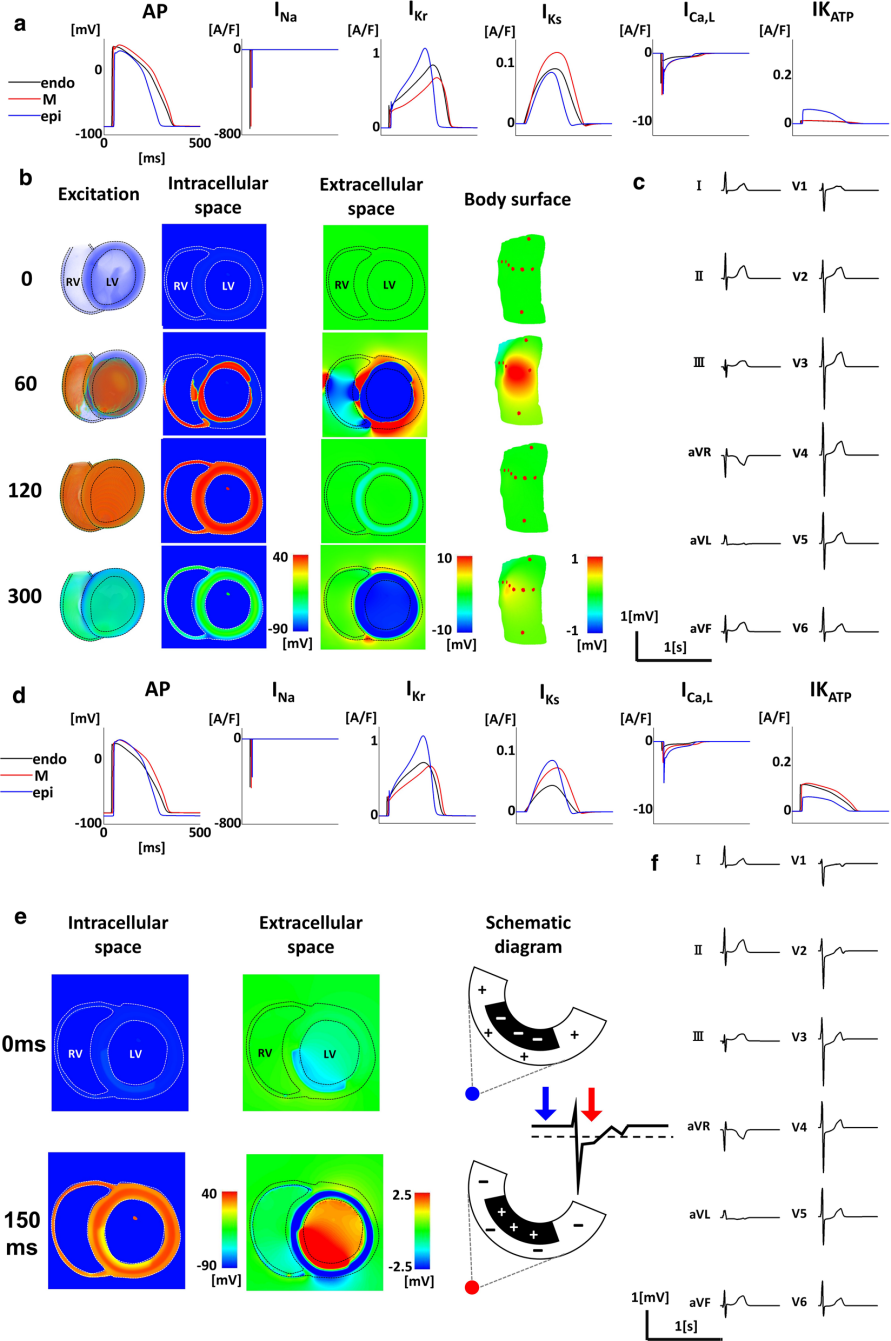
Electrophysiology under normal condition and subendocardial ischemia. **a** Cellular electrophysiology: Action potentials (AP) and major ionic currents of the endocardial (endo), mid-myocardial (M), and epicardial (epi) cells. **b** Time-lapse images showing propagation of excitation in the heart (Excitation), short axis views of the potential distributions in the intracellular space and extracellular space, and the body surface potential map. Numbers indicate the time after the onset of excitation in ms. RV, right ventricle; LV, left ventricle. **c** 12-lead electrocardiogram. **d** Cellular electrophysiology under subendocardial ischemia: AP and major ionic currents of the endo, M, and epi cells. **e** Short axis views of the potential distributions in the intracellular and extracellular spaces during diastole (0 ms) and systole (150 ms) under subendocardial ischemia. Electrical bilayers and their solid angles at the overlying electrodes are shown in the schematics (right column). RV: right ventricle; LV: left ventricle. **f** 12-lead electrocardiogram. INa, sodium current; IKr, rapid delayed rectifier potassium current; IKs, slow delayed rectifier potassium current; ICaL, L-type calcium current; IK_ATP_, ATP-sensitive potassium current

The electrophysiology of the heart under normal conditions and subendocardial ischemia were compared (Fig. 2). Under normal conditions, the action potential duration differed between the endocardial, M, and epicardial cells because of the differences in potassium (IKr and IKs) and calcium (ICaL) currents. The ATP-sensitive potassium current was completely inhibited transmurally (Fig. 2a). Ventricular activation is shown by the time-lapse images (Fig. 2b, left column; Additional file 1: Video S1). The short axis views at the mid-ventricular level clearly indicate that the membrane depolarization of the myocytes propagates from the endocardial surface to the epicardial side (Fig. 2b, intracellular space), while creating potential distributions opposite in polarity in the extracellular space (Fig. 2b, extracellular space). The extracellular potential distributions in the heart domain were reflected to the body surface potential (Fig. 2b, body surface), from which we could obtain the 12-lead ECG (Fig. 2c).

When the ischemic condition ([K]_o_ = 6.5 mM, [ATP] = 1.0 mM) was applied to the subendocardial region, the resting membrane potential of the endocardium became less negative compared with that of the normal region (Fig. 2d, action potential (AP)), creating an opposite potential gradient in the extracellular space. This, in turn, produced a positive potential recorded by a precordial electrode during diastole (Fig. 2e, 0 ms). During systole, when the entire wall was excited, the potential gradient was reversed and the precordial electrode recorded a slightly negative voltage (Fig. 2e, 150 ms; Additional file 2: Video S2). However, according to the convention in ECG recording, which sets the TQ-segment at zero, the ST segment is pushed further downwards to the negative side. Accordingly, ST depressions were observed in the precordial leads overlying the ischemic region (Fig. 2f).

### ECGs under various severities of transmural ischemia

**Fig. 3:**
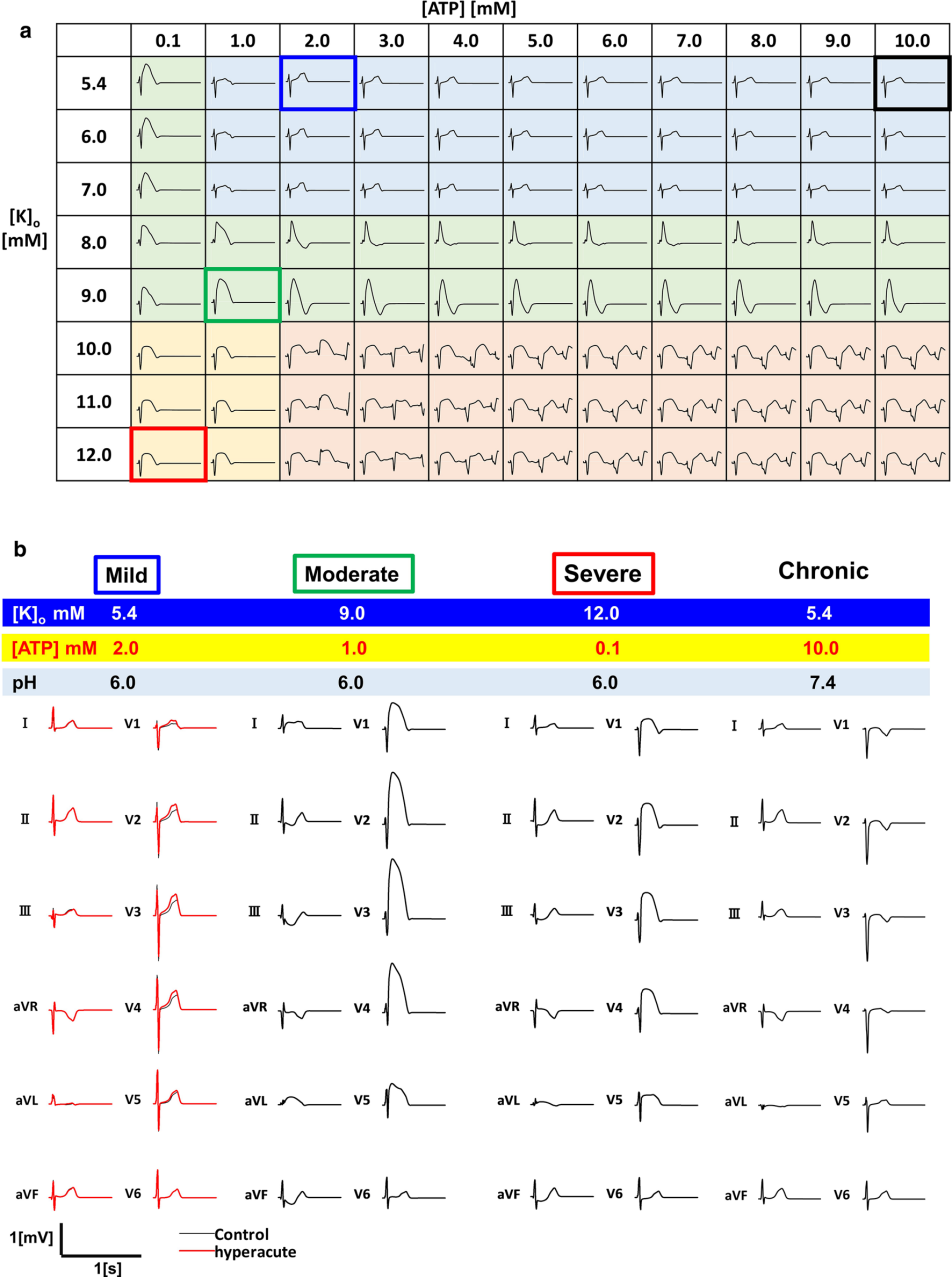
Effects of transmural hyperkalemia and adenosine triphosphate (ATP) depletion on the electrocardiogram (ECG). **a** Simulated V2 lead ECGs under various concentrations of ATP and potassium are shown. ECGs with similar morphologies are grouped with different colors (mild: light blue; moderate: green; severe: light brown; arrhythmia: dark brown). **b** 12-lead ECGs of representative cases are shown. Conditions were selected from (**a**) for normal (black rectangle), mild ischemia (blue rectangle), moderate ischemia (green rectangle), and severe ischemia (red rectangle)

The V2 lead ECGs under various severities of transmural ischemia are shown in Fig. 3a. Under conditions clustered in the upper right (blue), ECG morphology was largely similar to the normal pattern (black bold rectangle), although the amplitude of the T wave increased at 1 mM [ATP], and then decreased with further reductions in [ATP]. In the green colored region characterized by moderately increased [K]_o_ and/or severe depletion of [ATP], we observed prominent ST–T elevation resembling a tombstone morphology [5]. The pattern of horizontal ST elevation often described in textbooks appeared under severe hyperkalemia and ATP depletion (light brown). In the dark brown region, we observed ventricular arrhythmias.

**Fig. 4:**
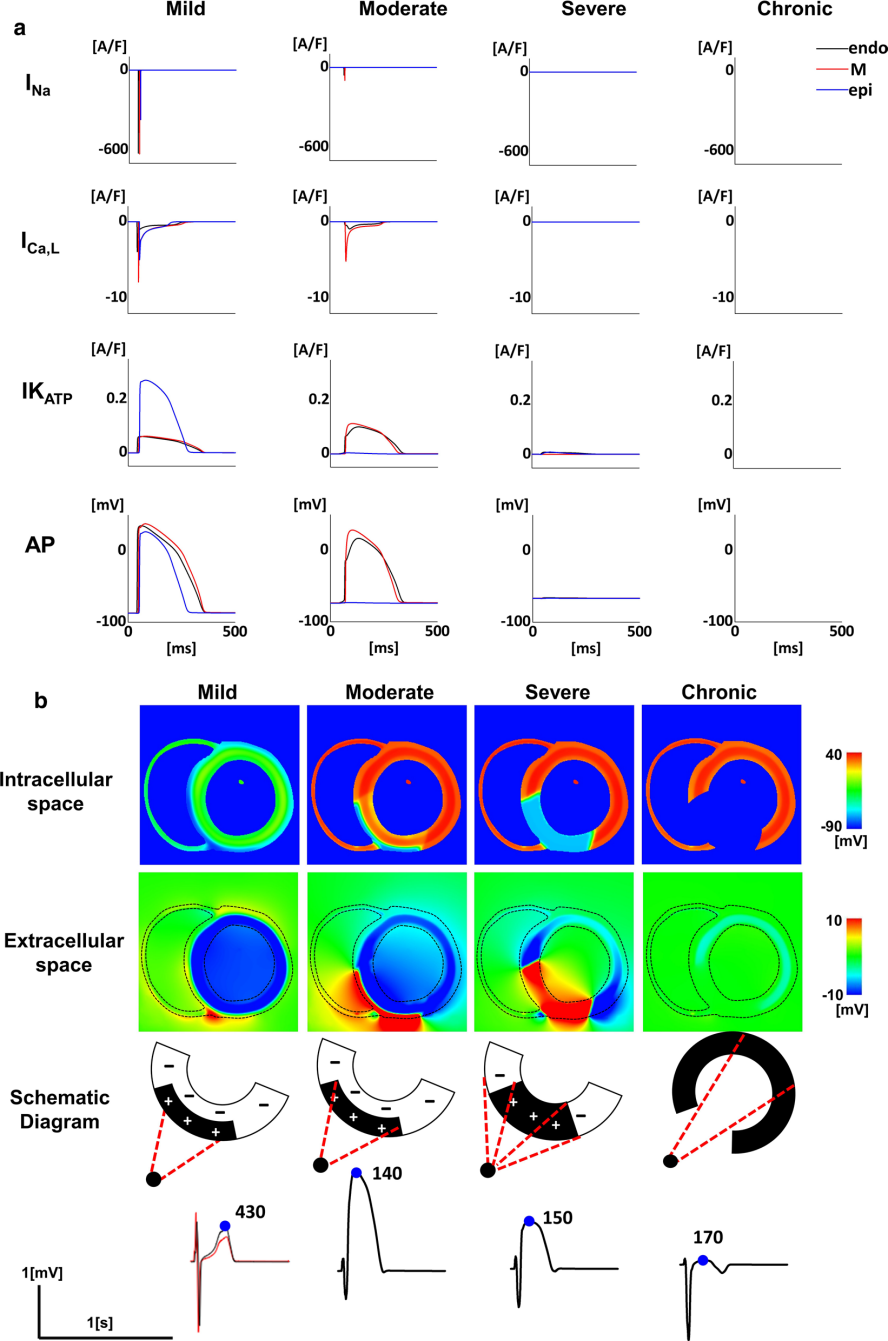
Mechanisms of ST–T changes under ischemic conditions. **a** The sodium current (INa), L-type calcium current (ICaL), rapid delayed rectifier potassium current (IKr), adenosine triphosphate (ATP)-sensitive potassium current (IK_ATP_), and action potential (AP) are shown under mild, moderate, and severe acute ischemia. During chronic ischemia, cellular activities were completely abolished. In each panel, data from the endocardial region are shown by a black line, the mid-myocardial region by a red line, and the epicardial region by a blue line. **b** The potential distributions in the intracellular (top row) and extracellular (second row) spaces (at the timing indicated by the blue dot in the below electrocardiogram [ECG]) are shown for mild, moderate, and severe ischemia in the acute and chronic phases. The electrical bilayers and their solid angles at the overlying electrodes are shown in the schematic (third row). The bottom panels show the ECGs (V3 lead) under each condition. The ECG under mild ischemia is compared with the normal ECG (red line). Numbers at the bottom indicate the time after the onset of ventricular activation in milliseconds

To elucidate the mechanisms underlying these ECG changes, we sampled and compared the three conditions marked by blue, green, and red rectangles (Fig. 3a), representing mild, moderate, and severe ischemia, respectively. Various waveforms were observed on the 12-lead ECGs during the three increasing severities of transmural ischemia and in the chronic phase, representing the course of myocardial infarction (Fig. 3b). The cellular-level events underlying these ECG changes are summarized in Fig. 4a. Under the mild ischemic condition, selective activation of the IK_ATP_ and downregulation of INa in the epicardium shortened the action potential duration of this region. As the severity of ischemia was increased (moderate ischemia), a further inhibition of INa suppressed the action potential in the epicardium. INa also started to decline in the M and endocardial cells, and with the resultant depression in ICaL the amplitude and duration of the action potentials of these cells were reduced. Under the severe ischemic condition, the action potentials were suppressed transmurally. In the chronic phase, the electrophysiological activities of the ischemic region were eliminated, and only the extracellular space was present transmurally.

To further elucidate how these cellular events were translated into ECG changes, we examined the potential distributions in the intracellular and extracellular spaces of the ventricular wall (Fig. 4b). Because we applied the same [K]_o_ (determinant of resting membrane potentials) transmurally, only the systolic phase was examined. Under mild ischemia, an electrical bilayer, which appeared in the extracellular space during the late systole, caused broad-based, tall T waves (mimicking the so-called hyper-acute T waves) on the electrode overlying the ischemic region (Additional file 3: Video S3). Moderate ischemia selectively suppressed the action potential of the epicardial layer (intracellular space), thus creating an electrical bilayer with a large magnitude and an opposite polarity to that observed during subendocardial ischemia (Fig. 2d) throughout systole (Additional file 4: Video S4). The resulting prominent ST elevation resembled the tombstone morphology reported in clinical and experimental acute ischemia [6]. The further increase in severity (severe ischemia) suppressed the action potentials transmurally (intracellular space), although the bilayers still existed at the boundaries between the ischemic and non-ischemic regions, which generated an ST elevation of a moderate magnitude (Additional file 5: Video S5). Finally, in the case of chronic infarction, the precordial electrode measured the potential created by the posterolateral wall from inside the ventricle, with a resulting inverted ECG waveform (Additional file 6: Video S6).

### Mechanisms of arrhythmia

**Fig. 5:**
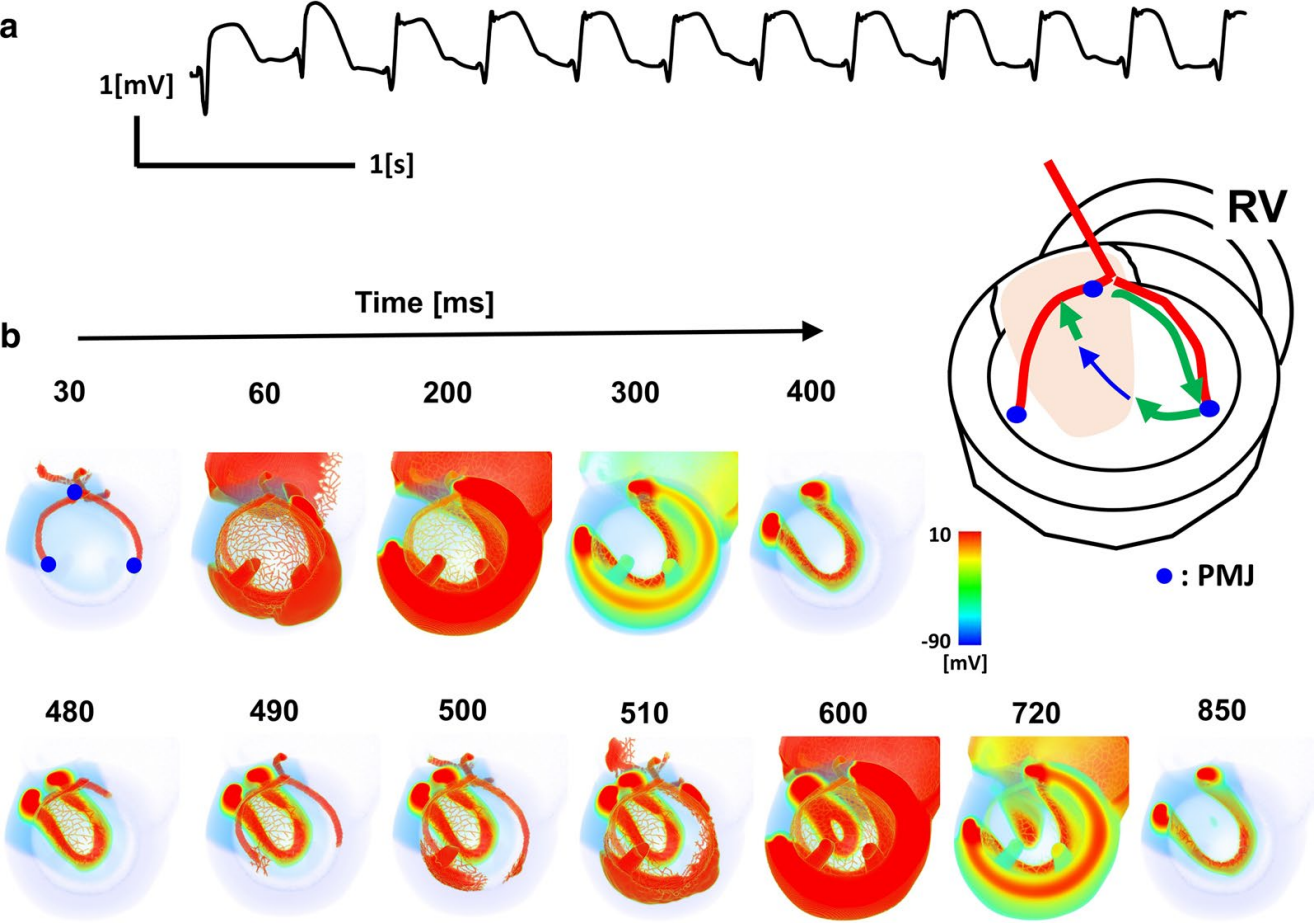
Ventricular arrhythmia during transmural ischemia. **a** V3 lead electrocardiogram (ECG). **b** Time-lapse images of ventricular activation viewed from the top. The numbers indicate the time after onset in ms. Inset: schematic presentation of the reentrant circuit. Excitation transmitted from the His–Purkinje system (red lines) propagates rapidly in the normal myocardium (green arrow), propagates slowly in the ischemic region (light brown; thin blue arrow), and finally back-propagates to the His–Purkinje system. PMJ: Purkinje–muscle junction

Arrhythmias were observed (Fig. 5a) under hyperkalemia with preserved ATP concentration ([ATP]) (light brown; Fig. 3a). Under these conditions, excitation initiated in the normal myocardium propagated slowly in the ischemic region (20–400 ms; Fig. 5b), then back-propagated to the already repolarized region via the Purkinje network (480–600 ms; Fig. 5b) to establish a reentry circuit (Fig. 5b, inset). Thus, slow conduction in the ischemic region may play a key role in the development of arrhythmias (see also Additional file 7: Video S7).

## Discussion

### Mechanisms of ST changes in ischemia

ST elevation is a hallmark of myocardial infarction caused by transmural ischemia. However, the ionic mechanism is less understood compared with the ST depression observed during subendocardial ischemia. Because ST depression is caused by the ventricular gradient directed towards the epicardium, the reversed gradient has been postulated as the cause of ST elevation [10, 12]. However, considering the favorable anatomy and physiology of epicardial tissue for coronary perfusion [18], such a situation is unlikely to occur. In the present study, using a multi-scale heart simulation, we examined whether such a gradient is possible, and if so, how it contributes to the genesis of ST elevation.

We postulated that the transmural heterogeneity in the properties of sodium and ATP-sensitive potassium channels [4, 8] would cause the transmural gradient, which would be dependent on ischemia severity. We found that the properties of these channels can create a ventricular gradient under moderately ischemic conditions, while under severely ischemic conditions, ST elevation was observed without the transmural gradient. As summarized (Fig. 3), under the ATP-depleted condition, an increase in potassium concentration changed the ECG morphologies from broad-based, tall T waves mimicking the so-called hyper-acute T [14], to a tombstone-like morphology [6], and finally to horizontal ST elevation. Because potassium homeostasis was maintained by the ATP-dependent *I*_NaK_ current, hyperkalemia develops after ATP depletion. Therefore, the observed changes in ECG morphologies showed similarities to the clinical course of transmural ischemia.

Under mild ischemia, we observed broad-based, tall T waves mimicking the so-called hyper-acute T waves, although the amplitudes were not high. We tested several parameters, and found that an increase in the Kd of ATP for the K_ATP_ channel (Kd_KATP_) in the epicardial cell was able to reproduce the hyper-acute T waves typically reported in the literature [14] (Additional file 8: Fig. S1). In the present study, because there were no reported experimental human data, we used the value reported by Furukawa et al. [8]. However, the actual value may differ in human ventricular tissue. However, the actual value may differ in human ventricular tissue.

### Solid angle theory and electrocardiogram

Quantitative analysis of ECG requires the computation of potential distribution in the torso domain with a complex structure and a distribution of conductivity. However, a qualitative explanation can be achieved using solid angle theory, by which the potential produced by a uniform electrical double layer is equal to the products of the double-layer density and the solid angle of the double layer surface viewed from the overlying electrode [7]. This theory is widely used for analysis of ECG, and its utility has been confirmed experimentally [10, 25]. Nevertheless, as that study assumed that the gradient in the intracellular space was the source of the double layer, these authors needed to locate the ischemic region on the epicardial side to explain the genesis of ST elevation [10]. As discussed, the body surface potential is generated by current flowing in the extracellular space, while the gradient in the extracellular space, but not in the intracellular space, can explain the ST elevation in transmural ischemia.

**Fig. 6:**
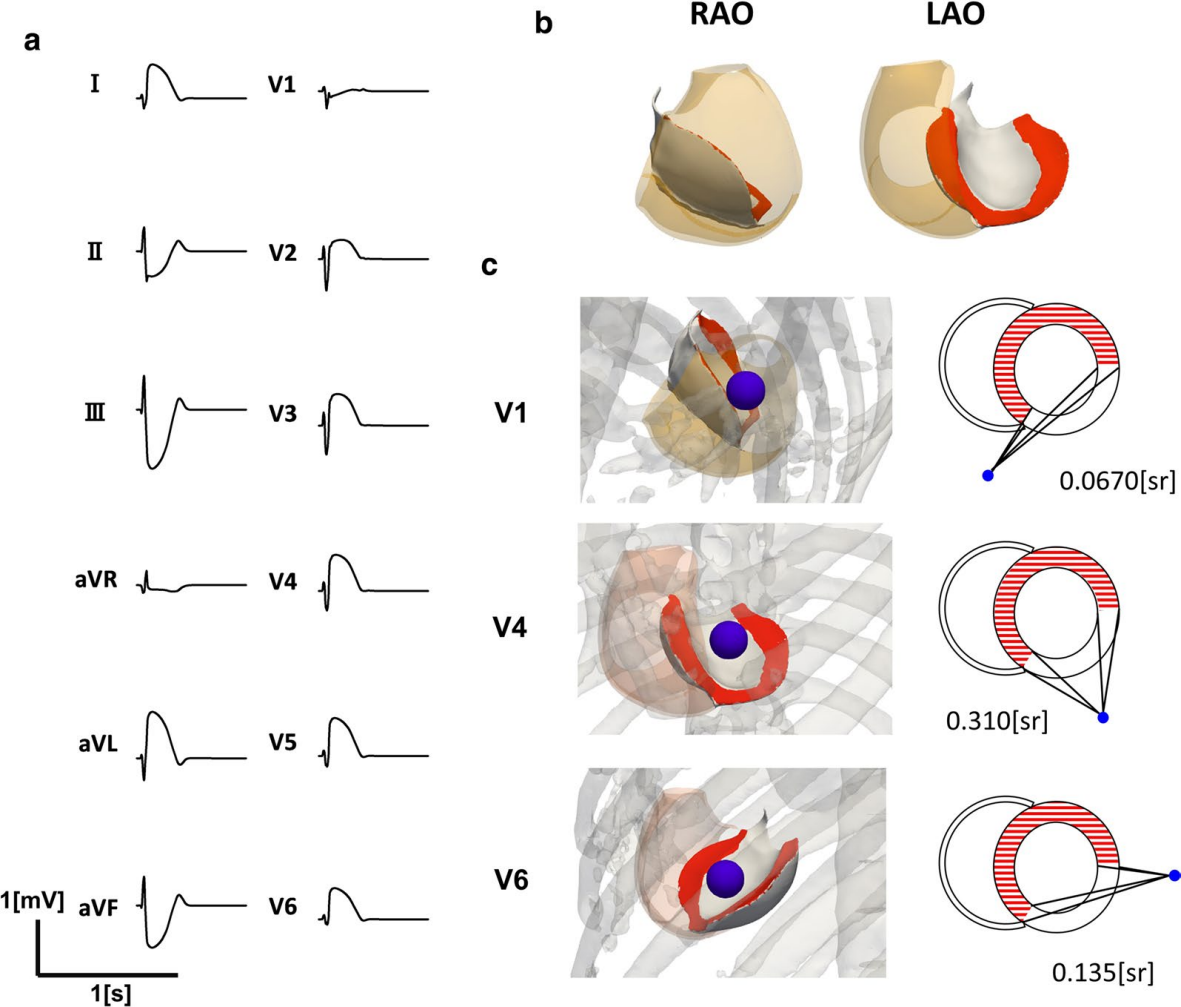
Magnitude of ST elevation and the lead position. **a** Electrocardiogram of the antero-lateral myocardial infarction. **b** The area of infarction in the right anterior oblique (RAO) and left anterior oblique (LAO) views. The area of infarction was extended to the lateral wall. The red areas indicate the boundary between the ischemic and non-ischemic myocardium. **c** Boundaries between the normal and infarcted area (indicated in red) viewed from the V1, V4, and V6 leads. In each panel, the schematic representation of the solid angle is shown on the right side. Non-ischemic areas are indicated by red stripes. Numbers indicate solid angles for the ischemic boundaries

For instance, ST elevation in moderate ischemia is much greater compared with that during severe ischemia because the solid angle of the bilayer is larger. Furthermore, solid angle theory can explain the electrode-position-dependent changes in the magnitude of ST elevation. The simulated ECG obtained from the model is shown in Fig. 6a, with an area of myocardial infarction extending to the lateral wall (Fig. 6b). The magnitude of the ST elevation peaks in the V3 and V4 leads, and gradually reduces towards the V1 and V6 leads, because the magnitudes are proportional to the solid angle of the boundary (red area, Fig. 6b) viewed from the corresponding electrodes.

There are some limitations of the present study. First, although our multi-scale heart simulator using widely adopted cell models of electrophysiology [17, 28] has successfully reproduced the ECG of healthy and diseased human hearts [19–21], other factors not included in our model may play key roles in the genesis of ECG during ischemia. For instance, intracellular Na^+^ increases two- to fivefold after 10–20 min of ischemia, via inhibition of the Na^+^–K^+^ pump and inward leak of Na^+^ (e.g., through the Na^+^/H^+^ exchanger). Upon reperfusion, increased intracellular Na^+^ stimulates the Na^+^–K^+^ pump, and eventually activates outward K^+^ currents. The resulting hyperpolarization of the membrane potential and shortening of the action potentials lead to occurrence of arrhythmias. Ischemia also increases the Ca^2+^ concentration in the cytoplasm, sarcoplasmic reticulum, and mitochondria, which is mainly caused by less efficient removal by the Na^+^/Ca^2+^ exchanger and reduced uptake by the Ca^2+^ pump in the sarcoplasmic reticulum. In turn, this increase in intracellular Ca^2+^ can modulate the activity of many ion currents, including the I_Ca,L_, I_Ks_, I_Kr_, I_NaK_, and Na^+^/Ca^2+^ exchanger currents. Such changes in ionic currents also lead to the occurrence of arrhythmias [3]. Future studies are required to update our model in line with these findings. Our model should be updated in line with future findings. Second, because the purpose of this study was to elucidate the ionic mechanism of ST elevation, the conditions and mechanisms of arrhythmias were not fully examined. Further studies under a wider range of conditions, including the anatomical variations of the infarcted region, will improve our knowledge on the diagnosis of myocardial ischemia. Third, while the effects of ATP and K^+^ concentrations were simulated in a graded manner, the effect of pH was only studied at one value (pH = 6.0) because we were unable to find quantitative data applicable to the ion channel models under multiple pH conditions. Nevertheless, considering the metabolic changes observed during myocardial ischemia, this is a major limitation of our study, and should be addressed in future research. Finally, in addition to the time-dependence of the severity of ischemia, it is important to consider the transmural gradient using more severe ischemia in the endocardial region. Because such a transmural ischemia gradient results in a higher K^+^ and lower ATP condition in the endocardium, which may negate the effect of the lower sensitivity of the K_ATP_ channel in the endocardium, we tested two cases of graded ischemia. When a severe (red rectangle in Fig. 3a) to moderate (green rectangle in Fig. 3a) gradient of ischemia was applied transmurally, we observed ST elevations that were similar to those under homogeneous severe transmural ischemia (Additional file 9: Fig. S2a). By contrast, a moderate to mild gradient produced ST depressions that were similar to subendocardial ischemia (Additional file 9: Fig. S2b). Although we used a limited range of conditions, the transmural gradient did not significantly alter the ST–T changes during ischemia.

## Conclusions

Transmural heterogeneities of the properties of sodium and ATP-sensitive potassium channels may contribute to the genesis of ST–T changes during mild or moderate transmural ischemia, while ST elevation may be induced without the contribution of heterogeneity under severe transmural ischemia. These results provide the comprehensive understanding of the ionic mechanisms of ECG changes during transmural ischemia.

### Supplementary information

**Additional file 1: Video S1.** Ventricular activation under normal conditions. Upper left: membrane potential in the ventricles. Upper right: body surface potential. Lower left: potential distribution in the intracellular space. Lower right: potential distribution in the extracellular space.

**Additional file 2: Video S2.** Ventricular activation during subendocardial ischemia. Left: membrane potential in the ventricles. Middle: potential distribution in the intracellular space. Right: potential distribution in the extracellular space.

**Additional file 3: Video S3.** Ventricular activation during transmural mild ischemia. Left: membrane potential in the ventricles. Middle: potential distribution in the intracellular space. Right: potential distribution in the extracellular space.

**Additional file 4: Video S4.** Ventricular activation during transmural moderate ischemia. Left: membrane potential in the ventricles. Middle: potential distribution in the intracellular space. Right: potential distribution in the extracellular space.

**Additional file 5: Video S5.** Ventricular activation during transmural severe ischemia. Left: membrane potential in the ventricles. Middle: potential distribution in the intracellular space. Right: potential distribution in the extracellular space.

**Additional file 6: Video S6.** Ventricular activation under transmural ischemia in the chronic phase. Left: membrane potential in the ventricles. Middle: potential distribution in the intracellular space. Right: potential distribution in the extracellular space.

**Additional file 7: Video S7.** Ventricular arrhythmia during transmural ischemia.

**Additional file 8: Figure S1.** Effect of KdKATP channel expression level on T wave morphology. (a) Action potentials of endocardial, M-, and epicardial cells under mild ischemia ([K]o = 5.4 mM, adenosine triphosphate [ATP] = 2.0 mM, and pH 6.5) in the model, in which the expression of the KdKATP channel in the epicardial cell was 0.1 mM (top) or 0.3 mM (bottom). (b) Electrocardiograms (ECGs) under control condition (black line), mild ischemia with KdKATP = 0.1 mM (blue line), and mild ischemia with KdKATP = 0.3 mM (red line).

**Additional file 9: Figure S2.** Effect of the transmural gradient of ischemia severity. (a) Epicardial and M- cells: [ATP] = 1.0 mM, [K]o = 9.0 mM, pH = 6.0; Endocardial cell: [ATP] = 0.1 mM, [K]o = 12.0 mM, pH = 6.0. (b) Epicardial and M- cells: [ATP] = 2.0 mM, [K]o = 5.4 mM, pH = 6.0; Endocardial cell: [ATP] = 1.0 mM, [K]o = 9.0 mM, pH = 6.0.

## Data Availability

The data that support the findings of this study are available from the corresponding author upon reasonable request.

## Abbreviations

ECG: Electrocardiogram
ATP: Adenosine triphosphate
M: Mid-myocardial
RAO: Right anterior oblique
LAO: Left anterior oblique
