# Supplementary figures and images for: Ionic mechanisms of ST segment elevation in electrocardiogram during acute myocardial infarction
Source: J Physiol Sci. 2020 Jul 13;70:36. doi: 10.1186/s12576-020-00760-3 (PMC10717899; doi:10.1186/s12576-020-00760-3)

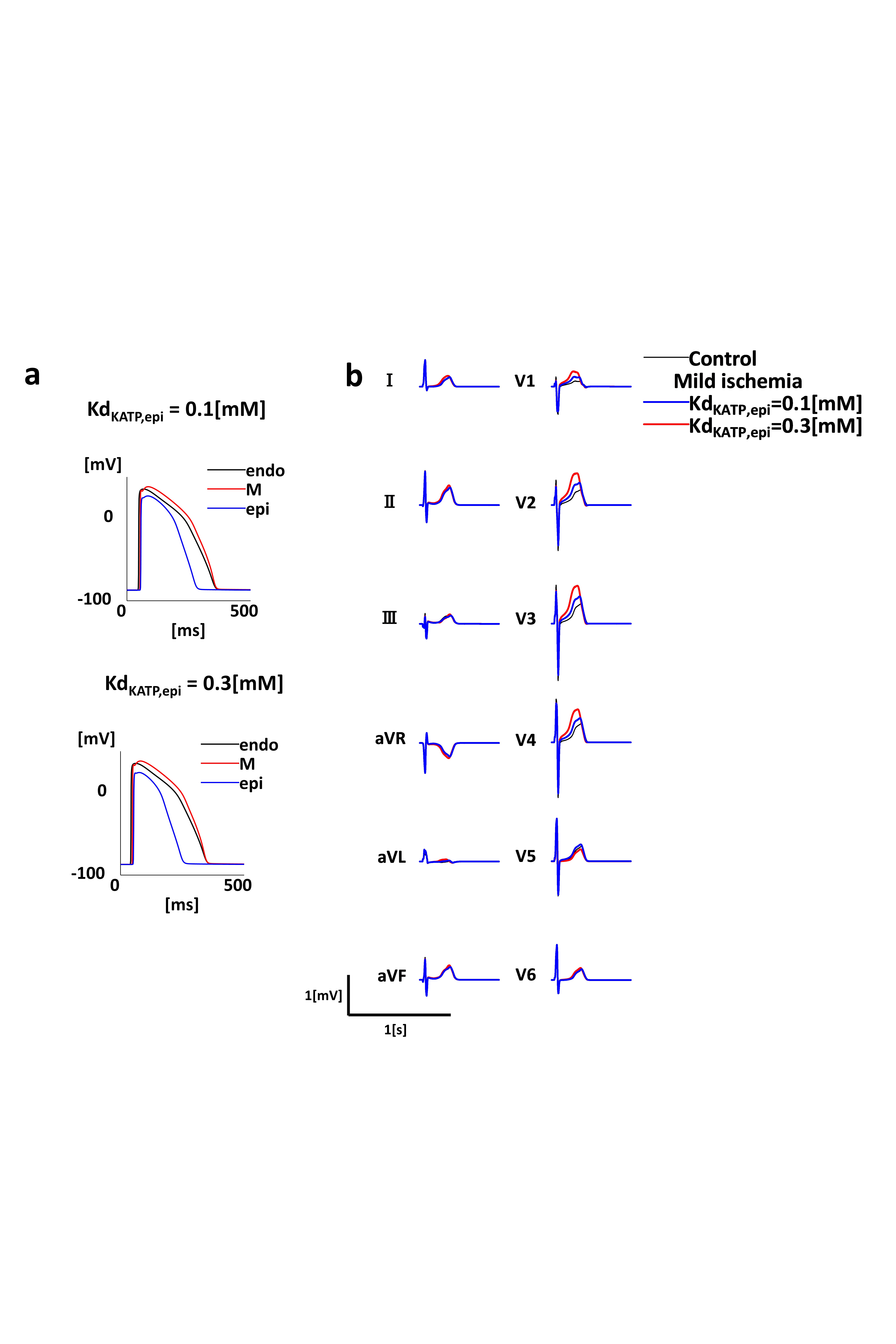

Supplement: Supplementary file 8 — Additional file 8: Figure S1. Effect of KdKATP channel expression level on T wave morphology. (a) Action potentials of endocardial, M-, and epicardial cells under mild ischemia ([K]o = 5.4 mM, adenosine triphosphate [ATP] = 2.0 mM, and pH 6.5) in the model, in which the expression of the KdKATP channel in the epicardial cell was 0.1 mM (top) or 0.3 mM (bottom). (b) Electrocardiograms (ECGs) under control condition (black line), mild ischemia with KdKATP = 0.1 mM (blue line), and mild ischemia with KdKATP = 0.3 mM (red line). [file 12576_2020_760_MOESM8_ESM.jpg]

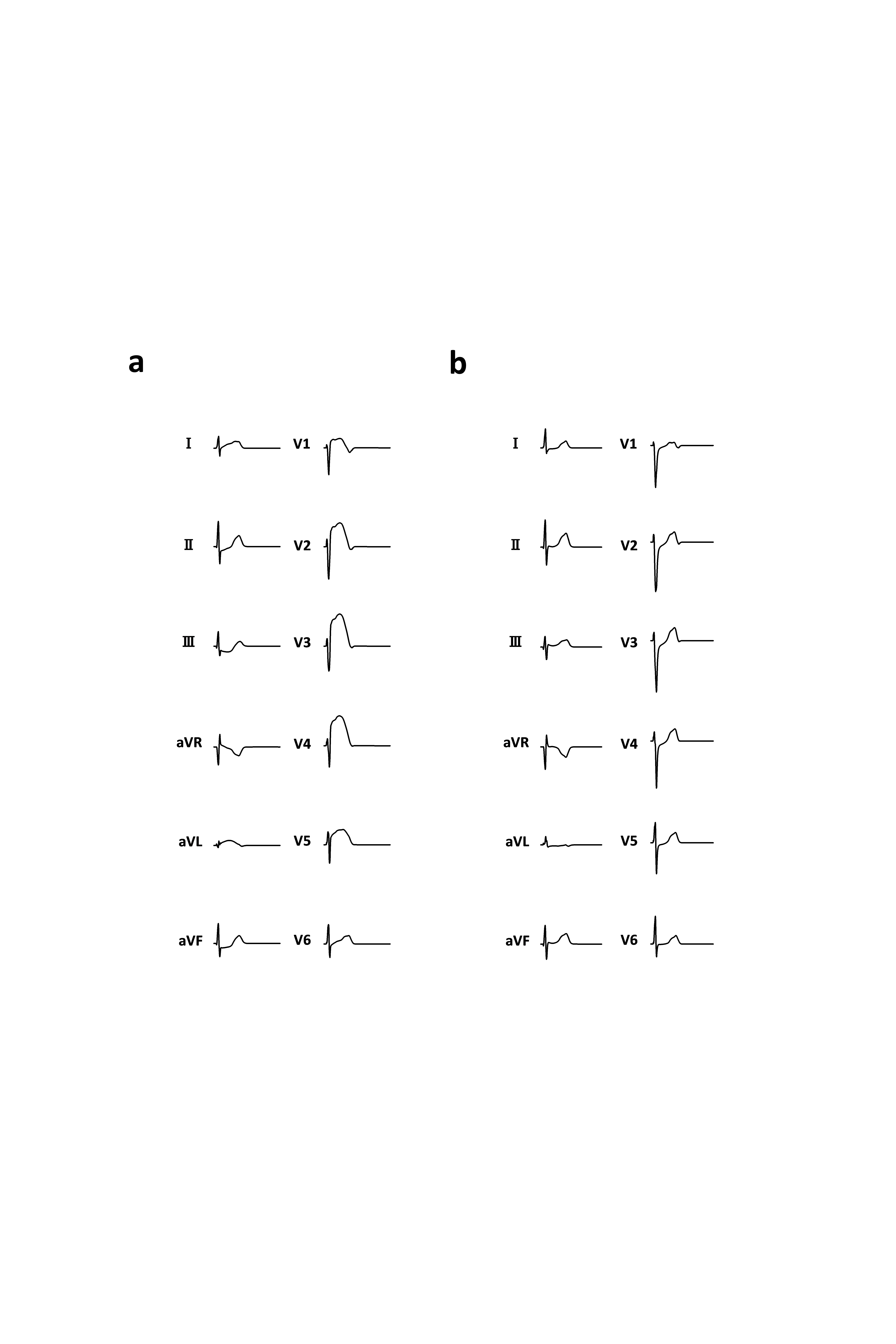

Supplement: Supplementary file 9 — Additional file 9: Figure S2. Effect of the transmural gradient of ischemia severity. (a) Epicardial and M- cells: [ATP] = 1.0 mM, [K]o = 9.0 mM, pH = 6.0; Endocardial cell: [ATP] = 0.1 mM, [K]o = 12.0 mM, pH = 6.0. (b) Epicardial and M- cells: [ATP] = 2.0 mM, [K]o = 5.4 mM, pH = 6.0; Endocardial cell: [ATP] = 1.0 mM, [K]o = 9.0 mM, pH = 6.0. [file 12576_2020_760_MOESM9_ESM.jpg]
